# Validation of wearable vital signs monitoring: A comparison with conventional bedside patient monitors

**DOI:** 10.1177/20552076251377934

**Published:** 2025-11-03

**Authors:** Weiyi Jiang, Wenzhao Zhang, Haoxuan Li, Jean Ngoie, Wenda Li, Zhihong Huang

**Affiliations:** 1 School of Physics, 8748 Engineering and Technology, University of York, York, UK; 2 Institute of Signals, Sensors and Systems, 3042 Heriot-Watt University, Edinburgh, UK; 3 CHEO Research Institute, 3120 Ottawa, Ontario, Canada

**Keywords:** Vital signs, digital health, newborn health, patient monitoring, wearables, wireless, Bland-Altman analysis

## Abstract

**Objective:**

This study aimed to assess how accurately mobile wearable devices could be used to monitor vital signs continuously in clinical settings by comparing their measurements with those from traditional bedside monitors.

**Methods:**

Data collected from Mindray's mWear wearable device were compared against measurements from the BeneVision N15 traditional bedside monitoring system. A total of 208 paired datasets, including blood pressure, oxygen saturation, heart rate, and respiratory rate, were collected from 16 healthy volunteers in a clinical setting. Bland-Altman analysis was applied to assess agreement between the two devices.

**Results:**

The analysis showed that 94.2% of the data variance points for oxygen saturation, diastolic blood pressure, and pulse rate fell within the limits of agreement. For systolic blood pressure, 92.3% of the data variance points were within limits, while 94.7% of the heart rate and respiratory rate data points were also within agreement limits.

**Conclusion:**

There was a strong agreement between the wearable mWear device and the traditional bedside patient monitoring system. This study supports the accuracy and reliability of wearable devices for continuous vital signs monitoring. These results encourage the wider use and ongoing improvement of wearable technology in clinical practice.

## Introduction

Currently, the National Health Service (NHS) in the United Kingdom relies on traditional bedside monitoring equipment, which restricts patients to their beds for monitoring. Patients have to remove all monitoring devices when they need to get out of bed. This results in a temporary cessation of effective monitoring and causes significant inconvenience. These issues lead to increased workload for NHS staff and consume valuable hospital bed capacity. Wearable monitoring devices can effectively address these problems by allowing continuous monitoring even when patients are mobile, thereby reducing the workload on healthcare staff and optimising the use of hospital resources.

Traditional monitoring practices heavily depend on bedside monitoring systems, which present significant challenges, particularly for low-income countries or healthcare institutions with limited resources. These systems are not only expensive but also impose a substantial burden on budgets. Additionally, their complex interfaces require specialised training, which increases the workload for healthcare professionals.^1^ Furthermore, the lack of portability due to their large size impedes their usability in various settings.

Recently, innovative advancements in patient vital signs monitoring have been boosted by smart wearable devices. These devices revolutionise healthcare by enabling continuous monitoring of vital signs and seamless connectivity via Bluetooth or Wi-Fi. This is especially valuable in scenarios requiring portability.^2,3^ Recognising the significance of this technology, the Royal College of Physicians updated its National Early Warning Score in 2017 to accommodate these new devices.^4–6^ In the United States, smart wearable adoption has reached 2%, and globally, the market is growing at a robust 25% annual rate. Forecasts indicate this trend will lead to a market share exceeding $70 billion by 2025.^7,8^

As the availability of smart wearable devices continues to expand, they are increasingly being utilised across various medical domains.^9,10^ However, a critical concern persists: do these devices offer comparable accuracy and validity to traditional bedside monitoring systems?^11^ While wearable devices feature remarkable flexibility and the ability to provide continuous vital sign monitoring, ensuring the quality of the data they generate poses a significant challenge in clinical settings. Achieving the requisite standard of accuracy and validity remains a hurdle for these devices. Despite numerous experiments and tests conducted on wearable technology, a notable gap exists in evaluating their effectiveness in real-world clinical applications.

In this article, we conduct a concurrent validity assessment of the mWear device, using the Mindray bedside patient monitoring system as the benchmark. The mWear, also developed by Mindray, is a wearable patient monitor specifically engineered to capture patients’ vital signs within hospital settings. Distinguished by its compact and lightweight design, mWear stands apart from conventional vital sign monitors, which typically comprise larger medical devices.

The primary objective of this article is to validate the comparability of the mWear device in monitoring four key vital signs—blood pressure, oxygen saturation, heart rate (HR), and respiratory rate (RR)—by comparing the vital sign data obtained from both the mWear device and the conventional monitoring system. Additionally, this study aims to evaluate the potential of the mWear device for adoption in the clinical setting.

Previous studies have also utilised Bland-Altman analysis to evaluate the agreement between wearable and clinical monitoring systems. For instance, Nantume et al. applied this method to assess a wireless vital signs monitor in a low-resource hospital setting, demonstrating its potential for clinical use despite certain limitations.^12^ This study builds on this approach by incorporating both simulator-based and human-subject data to provide a more comprehensive validation framework.

While previous studies have explored the use of wearable vital-sign monitors in low-resource settings, most focused on a limited number of physiological parameters and did not address practical implementation challenges in clinical settings.^13^ For instance, Garbern et al. conducted a clinical pilot study in Rwanda to evaluate a wearable biosensor for HR and RR only, and although the device showed good accuracy and early deterioration detection, they also reported issues such as intermittent connectivity, technical failures, and limited generalizability due to the single-site design.^11^

Similarly, Kaufmann et al.^14^ validated consumer wearables like the Fitbit Versa 2 and Xiaomi Mi Smart Band 6 in a Ghanaian hospital using Bland-Altman analysis, but their evaluation was limited to HR monitoring in healthy adults, lacking broader physiological assessment and clinical diversity.

Moreover, a recent systematic review has emphasised persistent challenges in clinical adoption of wearables, including inconsistent accuracy in blood pressure and RR, high rates of data loss, lack of integration with hospital IT infrastructure, and limited user acceptability in real-world settings. Additional studies from Uganda and Rwanda have documented infrastructural and cultural barriers such as unreliable power supply, limited internet access, and patient compliance issues, all of which hinder the deployment of such devices in low-resource healthcare environments.^15^

Importantly, these existing works tend to evaluate devices based on one or two vital signs in isolation, rather than assessing a full set of clinically relevant parameters (e.g. SpO₂, blood pressure, HR, pulse rate (PR), and RR) simultaneously, as is required in standard clinical practice. In contrast, our study provides a more comprehensive validation by examining multiple vital signs at once, using both simulator data and real human volunteer data, thereby offering a broader and more realistic assessment of wearable device concurrent validity in conditions that approximate real-world clinical use.

To highlight the gap in existing wearable technologies and to justify the choice of the mWear system, we briefly summarise several representative FDA-approved wearable monitoring devices below.

Furthermore, by contextualising our study with an overview of other established wearable devices, we aim to highlight the strengths and potential areas for improvement relevant to the clinical utility of such technologies. By doing so, it aims to provide valuable insights for healthcare professionals and patients contemplating the integration of wearable devices into medical practice.

The remainder of this article is structured as follows: the second section provides an overview of current advancements in wearable device technology and their development. The third section outlines the specifications of the devices employed in this investigation. The fourth section delineates the methodology employed to assess the concurrent validity of the wearable devices under scrutiny. The fifth section presents the findings derived from the experimental analysis. The sixth section engages in a discussion of the results obtained, as well as highlighting the limitations of this study. Finally, the seventh section offers concluding remarks.

## Background: Limitations of existing wearable devices

This section describes several FDA-approved wearable devices and demonstrates what they can achieve in modern medicine.

### Apple Watch

The Apple Watch, one of the most widely used wrist-worn wearables, has received FDA clearance for its electrocardiogram (ECG) and SpO₂ monitoring functions. It uses a Photoplethysmography sensor and a single-lead ECG system to detect irregular heart rhythms such as atrial fibrillation.^16^ Clinical studies have shown that it can reliably estimate HR and energy expenditure in cardiovascular patients,^17^ with measurements consistent with conventional clinical methods.^18^

### Alivecor KardiaMobile

The KardiaMobile is a portable, FDA-cleared ECG device capable of detecting atrial fibrillation, bradycardia, and tachycardia. Clinical studies have shown it achieves high sensitivity (93%) and specificity (83%) in Atrial Fibrillation rhythm interpretation,^18^ and it has been used effectively for long-term cardiac monitoring in patients with palpitations or arrhythmias.^9,10^

### SmartCardia

SmartCardia is a wireless wearable device capable of monitoring ECG, HR, RR, and skin temperature, with real-time arrhythmia detection supported by a neural network-based algorithm. Recent studies have demonstrated high accuracy in detecting atrial fibrillation, with F1 scores exceeding 92% in both public and clinical ECG datasets.^19^

While these devices have demonstrated clinical potential, they are typically limited to monitoring one or two physiological signals. None of them currently offers the capability to continuously monitor a full set of vital signs in a clinical setting. This limitation further supports our choice of the mWear device, which was specifically selected for its ability to provide comprehensive, multi-parameter monitoring comparable to conventional bedside systems.

## Methodology

This section details the methodology employed for this validation study. We first describe the experimental equipment, including the mWear wearable system, the BeneVision N15 reference monitor, and the ProSim 8 simulator used for generating baseline data. Subsequently, we outline the study design, which encompasses both the simulation trial and the clinical trial with healthy volunteers. Finally, we present the procedures for data collection, processing and the statistical methods—primarily the Bland-Altman analysis—used to assess the agreement between the devices. The study was conducted in accordance with the Declaration of Helsinki, and approved by the School of PET Ethics Committee of University of York (reference number is Jiang20241121).

## Experimental equipment for this study

### Mindray mWear

The mWear device consists of a set of systems. The EP30 device, serving as the central data-pooling unit, is equipped with a ring-type oximeter that monitors blood oxygen and PR in real time, and also calculates the HR from the ECG information when the ECG signal is absent. The ES30 unit measures the ECG signal and uses a Bluetooth module to transmit data to the EP30. The BP20 is used to measure the patient's blood pressure, measurement intervals can be freely adjusted, and Bluetooth technology is also used to communicate data to the EP30.

The data collected by the EP30 device can be read in the display on its wristband and also uploaded to the Central Monitoring System (CMS) for centralised processing via a Wi-Fi module. It allows continuous 24-hour patient monitoring and data to be observed from a station that connects to the device, which may be away from the patient and ward.

**Figure 1.**
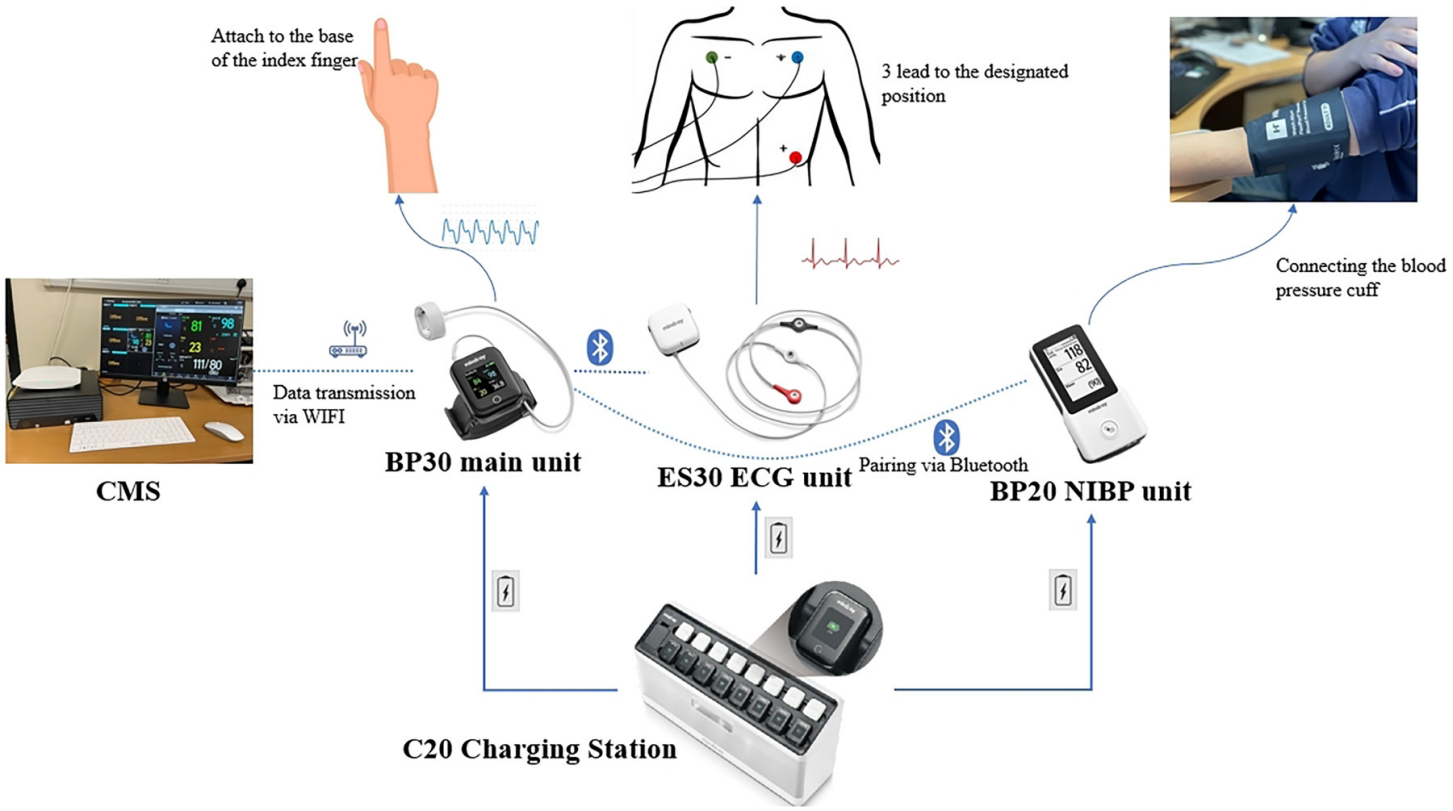
Overview of Mindray mWear system.

As a portable vital sign monitoring system, its mode of operation is illustrated in Figure 1. The EP30 and ES30's central processor and battery are integrated into a removable module that can be replaced at any time without affecting data connectivity, meanwhile the C20 charging station can provide power support for the spare module and the replacement module.

### BeneVision N15

The monitoring data from the BeneVision N15 system was used in this study as the reference data for comparison with the data from the mWear device. The BeneVision N15 is a clinically validated accurate and reliable bedside vital signs monitoring device.^20^

As a bedside monitoring device, the BeneVision N15 is widely used in hospitals, intensive care units, and operating theatres, where it plays an important role in both the intraoperative monitoring and postoperative care phases.^21–23^ Due to its proven technology and wide range of applications, it was selected in this study as a reference standard to compare the concurrent validity of mWear devices.

### ProSim 8 Simulator

Prior to conducting experiments on volunteers, the ProSim 8 Simulator was used to simulate human vital signs.

It can provide an accurate simulation of human vital signals in various movement states and can transmit the signals to the monitoring device through the sensors connected to the vital monitoring system. This has led to its use in a wide variety of studies that require the modelling of vital signals.^24^ In this study, the results of experiments conducted on the ProSim 8 Simulator will be used to compare with real human vital signals provided by volunteers.

### Study design

In this study, a bedside monitoring system from Mindray was chosen as the reference to validate the concurrent validity and comparability of Mindray's mWear device. The ProSim 8 Simulator was first used to simulate the vital sign signals of humans in different states of motion, and the signals from the simulator were monitored using the mWear and BeneVision N15 devices.

In selecting the devices for this study, we considered both functional and practical aspects. The wearable device needed to measure a full set of vital signs—including SpO₂, HR, RR, and both systolic (SBP) and diastolic blood pressure (DBP)—with clinical-grade accuracy. While some consumer wearables such as the Apple Watch or Fitbit offer partial monitoring capabilities (e.g. HR or SpO₂), they do not meet the comprehensive clinical requirements for multi-parameter monitoring. Therefore, the Mindray mWear was selected as it provides a complete set of vital sign measurements specifically designed for clinical use.

Similarly, the BeneVision N15 was selected as the reference device due to its widespread adoption in hospitals, its reliability, and its status as a clinically validated bedside monitoring system. This combination allowed for a meaningful and clinically relevant comparison between a modern wearable system and a standard hospital monitor.

**Figure 2.**
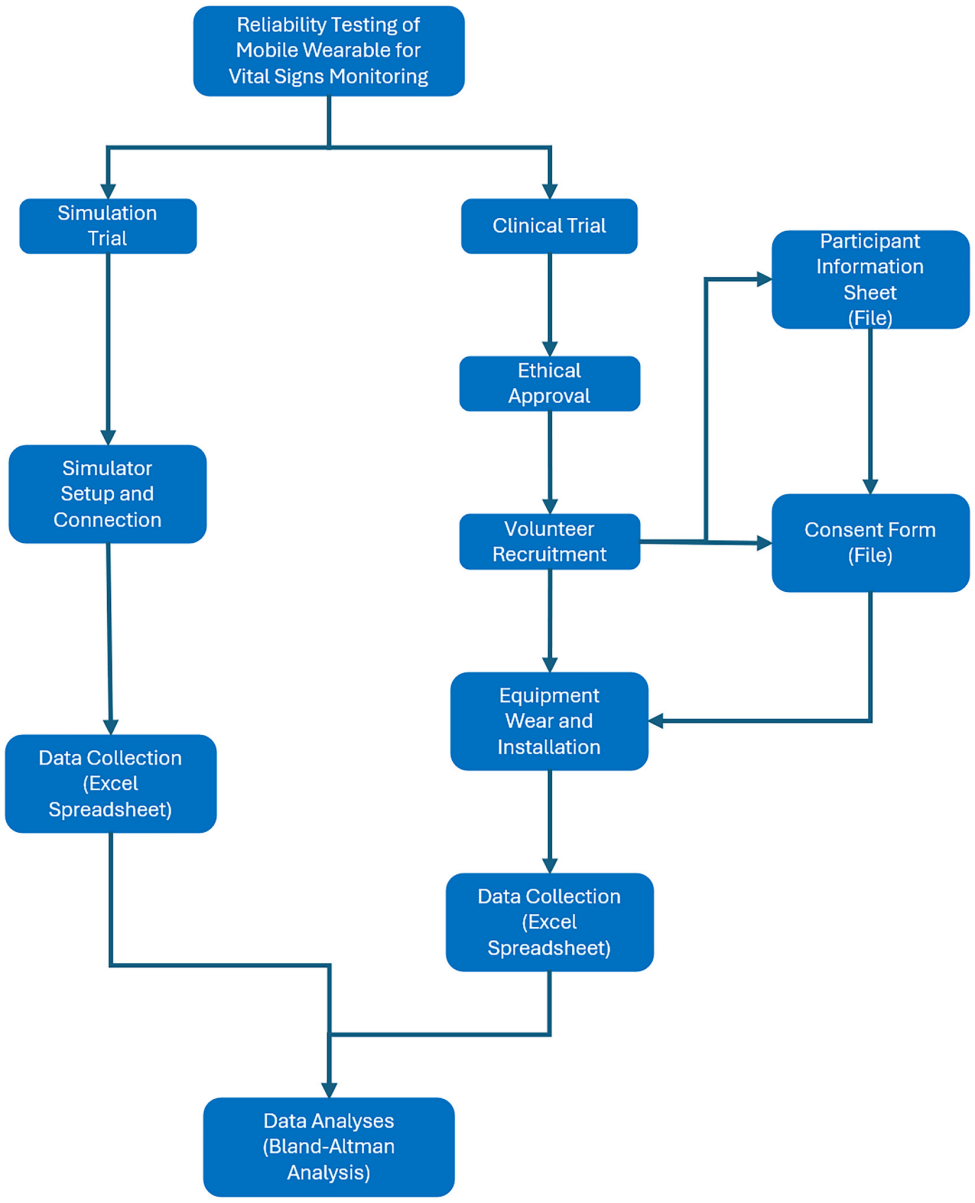
Experiment protocol.

As an alternative to simulation studies 16 apparently healthy volunteers between the ages of 21 and 30 years were recruited for a live study of the performance of the mWear devices. The specific experimental protocol is shown in Figure 2.

The subjects were monitored for a time period of 120 min with data collection at time 0 and 10 min. The study was conducted in accordance with the Declaration of Helsinki, and approved by the School of PET Ethics Committee of University of York (reference number is Jiang20241121). According to the requirements of the ethics approval, all volunteers who participated in this study signed the consent form and data collection form before the data collection.

The study was conducted at the Biomedical Engineering Lab, University of York between 2024 and 2025. The sample size was based on pilot-scale feasibility and technical validation, rather than powered statistical hypothesis testing. Inclusion criteria included: age between 18 and 60, no acute illness, and the ability to provide informed consent. Exclusion criteria included: diagnosed cardiovascular or respiratory disease, skin conditions affecting device attachment areas, pregnant or recent surgery.

Each volunteer wore both the mWear device and the reference bedside monitoring system, and the volunteer's peripheral capillary oxygen saturation (SpO_2_), non-invasive blood pressure (NIBP), PR, HR, and RR will be measured at the same time, with the measurement time set at 2 hours, and the data will be recorded at 10-minute intervals. Thirteen sets of data will be output for each vital sign of each volunteer.

### Data collection and processing

The structure of the data collection methodology was designed to provide a basis for rigorous Bland-Altman analyses,^25,26^ focusing on comparing data collected from the BeneVision N15 bedside patient monitor and the mWear patient monitor.

The monitoring data from the mWear device is read directly on the CMS monitor, and the data from the bedside monitoring system is read on the monitor that comes with the device. The focus of the study was to compare and reveal the differences between the data produced by the devices and to explore the data concurrent validity and application potential of the mWear device. The dataset for this study consisted of valid data points, basic data screening was conducted to exclude signal dropouts or motion artefacts before analysis, ensuring consistency across all devices.

The simulator's duration of continuous data collection was 1 hour, which generated a comprehensive dataset comprising no less than 3600 data points for each physiological parameter in five predefined patient conditions (normal, hypertensive, hypotensive, tachycardic, and bradycardic), each collected at one second intervals. To enhance the statistical robustness of the analyses, we further divided the data into sets with time intervals of minutes, thus creating a total of 60 subsets of clinical measurements for each patient monitor.

Following these steps, a comprehensive dataset encompassing 1200 pairs of measurements in total for SpO_2_, PR, ECG-derived HR, and RR was established across the five distinct patient conditions. Subsequently, the average values of these paired measurements were calculated and imported into MATLAB for the implementation of the Bland-Altman analysis.^12,26^

The duration of continuous data collection with volunteers was 2 hours. This generated a comprehensive dataset of each volunteer's vital signs, including SpO_2_, NIBP, PR, HR, and RR, with no less than 7200 data points for each physiological parameter.

To enhance the statistical robustness of the analyses, each volunteer's dataset was screened at 10-minute intervals for a total of 13 sets of data. A total of 208 sets of data were generated from 16 volunteers. These data were then imported into MATLAB to be analysed and plotted with Bland-Altman plots.^12,26^

### Statistical analysis

After data preprocessing, limits of agreement (LOA) was used to assess the agreement and accuracy of the data collected by the mWear device.^12^ For each parameter, the differences between paired measurements were calculated. The bias was determined as the mean of these differences. The 95% LOA were then calculated as the bias ± 1.96 times the standard deviation (SD) of the differences. The calculation is expressed by the following formula:

LOA=Bias±1.96*TotalSD

Before analysing the data, some target values were preset for the simulator experiments. For SpO_2_, we predefined a target range of LOA ≤ ± 3%; for PR, a predefined target range of LOA ≤ ± 2(bpm), for HR of ECG, a predefined target range of LOA ≤ ± 2(bpm); for RR of ECG, a predefined target range of LOA ≤ ± 2(brpm). These ranges are suggested by previous work.^12^

In the final phase of the experiment, a critical comparative analysis will be performed comparing the experimental results obtained on the simulator with those of the volunteer experiment. This is used to assess the agreement between the two sets of equipment.

## Results

### Results of data analysis

Bland-Altman analysis of the data using MATLAB produced experimental results for both the simulator experiment and the volunteer experiment.

Table 1 presents the summary statistics for the Bland-Altman analysis, detailing the measurements of SpO_2_, PR, HR, and RR. The analysis of all simulated physiological measurements consistently showed that the results remained within the predetermined LOA.

**Table 1.** Bland-Altman analysis summary statistics for simulator experiments.

	Normal	Hypertensive	Hypotensive	Tachycardic	Bradycardic
*Target LOA:* *±* *3%; and SpO_2_ actual values*					
*Bias*	*1.583*	*0.333*	*−0.667*	*0.617*	*−0.183*
*Total SD*	*0.855*	*0.261*	*0.429*	*0.624*	*0.600*
*Upper LOA*	*3.259*	*0.844*	*0.175*	*1.839*	*0.992*
*Lower LOA*	*−0.092*	*−0.177*	*−1.508*	*−0.605*	*−1.359*
*Target LOA:* *±* *2 bpm; and PR actual values*					
*Bias*	*−0.050*	*0*	*0.083*	*0*	*0*
*Total SD*	*0.193*	*0*	*0.180*	*0*	*0*
*Upper LOA*	*0.328*	*0*	*0.436*	*0*	*0*
*Lower LOA*	*−0.428*	*0*	*−0.270*	*0*	*0*
*Target LOA:* *±* *2 bpm; and HR actual value*					
*Bias*	*0*	*0*	*0*	*0*	*0*
*Total SD*	*0*	*0*	*0*	*0*	*0*
*Upper LOA*	*0*	*0*	*0*	*0*	*0*
*Lower LOA*	*0*	*0*	*0*	*0*	*0*
*Target LOA:* *±* *2 brpm; and RR actual values.*					
*Bias*	*0*	*0*	*0*	*0*	*0*
*Total SD*	*0*	*0*	*0*	*0*	*0*
*Upper LOA*	*0*	*0*	*0*	*0*	*0*
*Lower LOA*	*0*	*0*	*0*	*0*	*0*

HR: heart rate; LOA: limits of agreement; PR: pulse rate; RR: respiratory rate; SD: standard deviation.

This agreement between the observed LOA from Table 1 and the established target range confirms that the differences between the two measurement methods were within acceptable limits. The notable concordance highlighted in this study underscores the concurrent validity and uniformity of the measurements, aligning effectively with the predefined clinical or applied standards for acceptable agreement. As a result, researchers and clinical practitioners can confidently use either of the two methods interchangeably in various scenarios, assured that significant systematic differences are unlikely to pose a concern.

Table 2 represents the summary statistics of the Bland-Altman plot for volunteers with data on SpO_2_, SBP, DBP, PR, RR, and HR.

**Table 2.** Bland-Altman analysis summary statistics for volunteer experiments.

	SpO_2_	SBP	DBP	PR	HR	RR
*Bias*	*−1*.*034*	*−4*.*587*	*−0*.*495*	*−1*.*034*	*−0*.*168*	*1*.*423*
*Total SD*	*1*.*456*	*7*.*892*	*6*.*788*	*6*.*040*	*5*.*563*	*4*.*774*
*Upper LOA*	*1*.*820*	*10*.*882*	*12*.*809*	*10*.*806*	*10*.*736*	*10*.*779*
*Lower LOA*	*−3*.*887*	*−20*.*055*	*−13*.*800*	*−12*.*873*	*−11*.*073*	*−7*.*933*
*Consistent points*	*196*	*192*	*196*	*196*	*197*	*197*
*Deviant points*	*12*	*16*	*12*	*12*	*11*	*11*

DBP: diastolic blood pressure; HR: heart rate; LOA: limits of agreement; PR: pulse rate; RR: respiratory rate; SD: standard deviation; SBP: systolic blood pressure.

This method of analysis assesses the consistency of data obtained from the same patient when vital signs are measured with two devices simultaneously, and the Bland-Altman analysis is an effective means of assessing the consistency of measurements from a new device with those from an existing device, which aids in the assessment of bias and LOA.

### Bland-Altman plot

After monitoring data from 16 volunteers using the mWear device and the bedside monitoring device (BeneVision N15) simultaneously for vital signs, these 16 volunteers could produce a total of six types of data, where each type of data for each individual contained 13 sets of data, with a total of 208 pairs of data points for each type of data, and analysing each of the difference of those 208 pairs of data points can obtain six Bland-Altman plots.

### Result of SpO_2_

**Figure 3.**
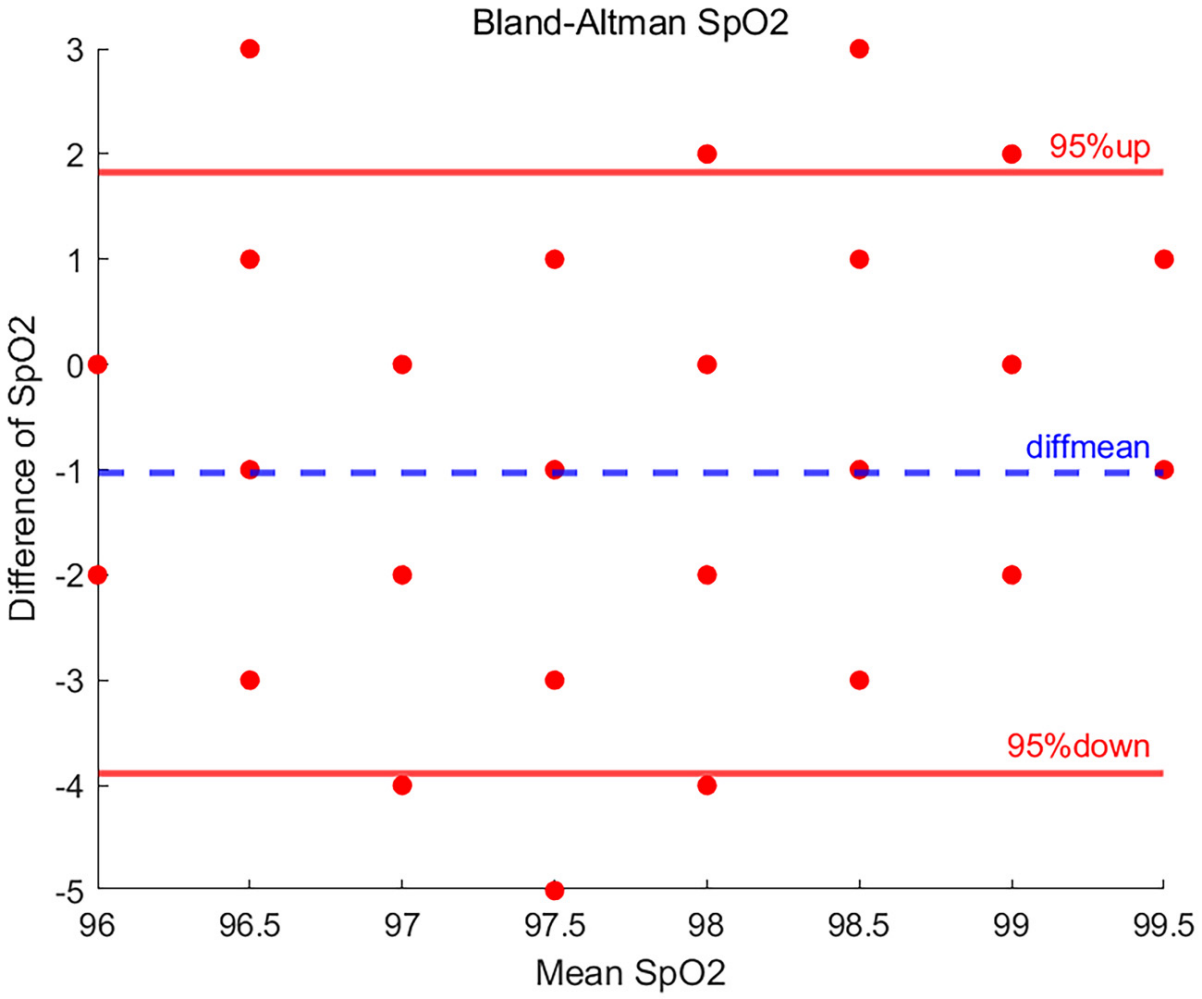
Bland-Altman plot of SpO_2_.

In this study, a total of 16 volunteers participated, generating 208 sets of blood oxygen saturation measurements. The results after Bland-Altman plot analysis are shown in Figure 3, which shows that 94.2% of the data points fell within the 95% LOA.

Specifically, this means that the vast majority of the differences between the measurements and the reference values are within an acceptable margin of error, indicating the agreement and accuracy of the EP30 device during SpO_2_ measurements. However, there were 12 data points where the difference was outside the 95% LOA, which may indicate an anomaly in individual measurements or potential systematic error. Across all SpO_2_ measurements, the SD of measurement differences was 1.456, with a mean difference of −1.034 indicating an overall slightly lower measurement value compared to the reference value.

In the Bland-Altman plot, the upper LOA interval is 1.820, while the lower LOA interval is −3.887, and the difference falling in this interval indicates that these data correspond to the accuracy and consistency of the SpO_2_ data measured by the EP30 device compared to the BeneVision N15.

### Result of systolic blood pressure

**Figure 4.**
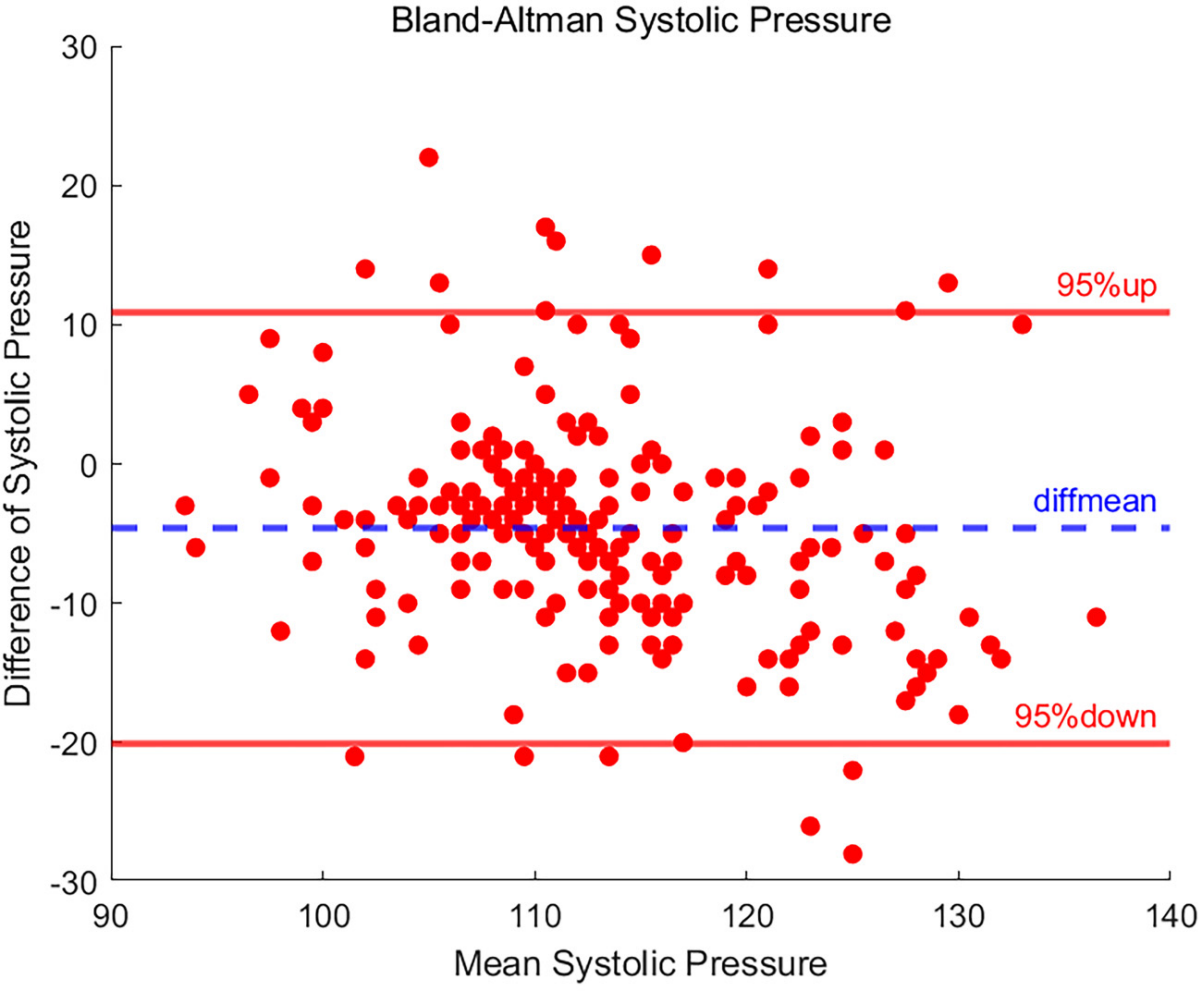
Bland-Altman plot of systolic blood pressure.

Blood pressure measurements were subdivided into SBP and DBP components for independent statistical analyses. For the 208 groups of SBP measurement data, the results after analysis by the Bland-Altman method are shown in Figure 4. And 92.3% of the SBP difference data fell within the 95% LOA, and this percentage reflects a high degree of agreement between the two measurement methods.

However, there were also 16 data points where the difference exceeded the upper and lower bounds of the calculated LOA, which may be indicative of individual measurement anomalies or potential systematic errors. Specifically, the SD of the difference in SBP data measured by the two devices was 7.892, with a mean difference of −4.587, which suggests that the SBP results obtained by the BP20 device were measured to be on the low side of the acceptable range.

The upper LOA interval determined by Bland-Altman analysis is 10.882, and the lower LOA interval is −20.055. Over 90% of the data points fall within this interval, demonstrating that in systolic pressure measurements, the systolic pressure data measured by BP20 is consistent with the reference value data measured by BeneVision N15.

### Result of diastolic blood pressure

**Figure 5.**
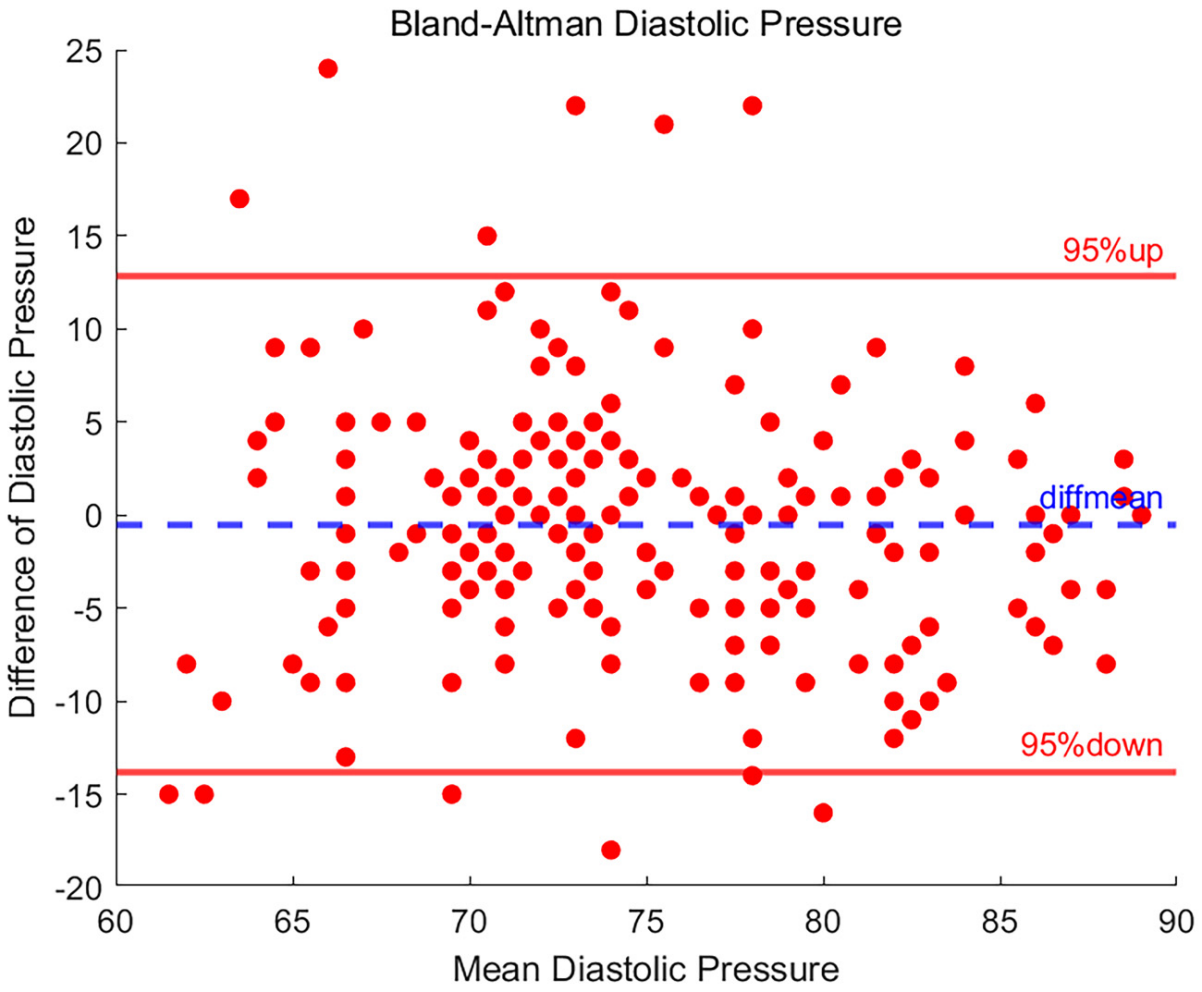
Bland-Altman plot of diastolic blood pressure.

In Figure 5, after separating the SBP and DBP data for independent statistical analysis, the DBP measurements were statistically analysed to assess agreement between the two different measurement devices.

Among the 208 sets of DBP data, there were 12 data points where the difference was outside of the 95% LOA, indicating that the difference between these measurements and the reference value was outside of the expected margin of error and may be indicative of an anomaly in the measurement process or the presence of a systematic error.

It is noteworthy that a total of 94.2% of the data points still fell within the 95% LOA for the difference, this percentage reflecting a more consistent measurement between the two measurements. The SD of the data difference for DBP was 6.788, a result that reveals less variability in measurements between the two devices than in measurements of SBP. The mean difference in DBP was −0.495, suggesting that the DBP measured by the BP20 demonstrated a slight negative bias when compared with BeneVision N15 device.

Bland-Altman analysis showed that the mean difference (bias) in DBP between the BP20 device and the BeneVision N15 reference was −0.495 mmHg, with 95% LOA ranging from −13.800 to 12.809 mmHg. This indicates that 95% of the measurement differences fall within this range, suggesting that there is no significant systematic bias between the two devices and that the differences are clinically acceptable.

### Result of pulse rate

**Figure 6.**
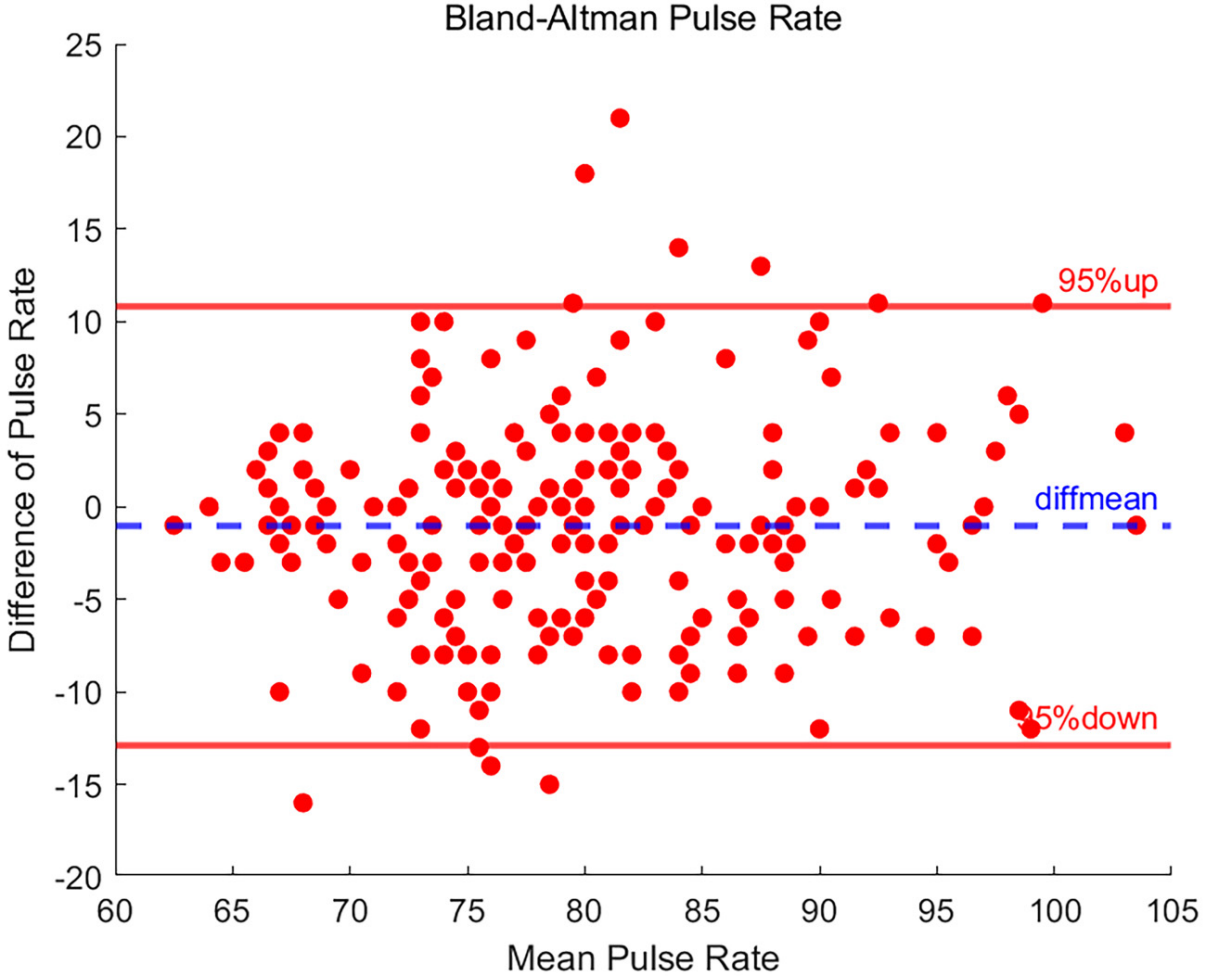
Bland-Altman plot of pulse rate.

The statistical results for PR are shown in Figure 6. Of the 208 sets of PR measurements, 12 sets of data points had differences outside the 95% LOA, indicating that the differences between these particular measurements and the reference values were outside the calculated margins of error, and may be indicative of anomalies or the presence of systematic errors in the measurement process.

However, overall, 94.2% of the data points still fall within the 95% LOA, reflecting a relatively consistent measurement result between the two devices. For the PR measurement data, the upper LOA and the lower LOA are 10.806 and −12.873, respectively. After calculation, the SD of the difference between the PR data is 6.040, and the average difference is −1.034. This result indicates that the PR measured by EP30 is slightly lower than the result of BeneVision N15.

From the results, 94.2% of the difference points fall within the consistency interval. This result indicates that the PR measured by EP30 has higher agreement and consistency compared to the reference data measured by BeneVision N15.

### Result of heart rate

**Figure 7.**
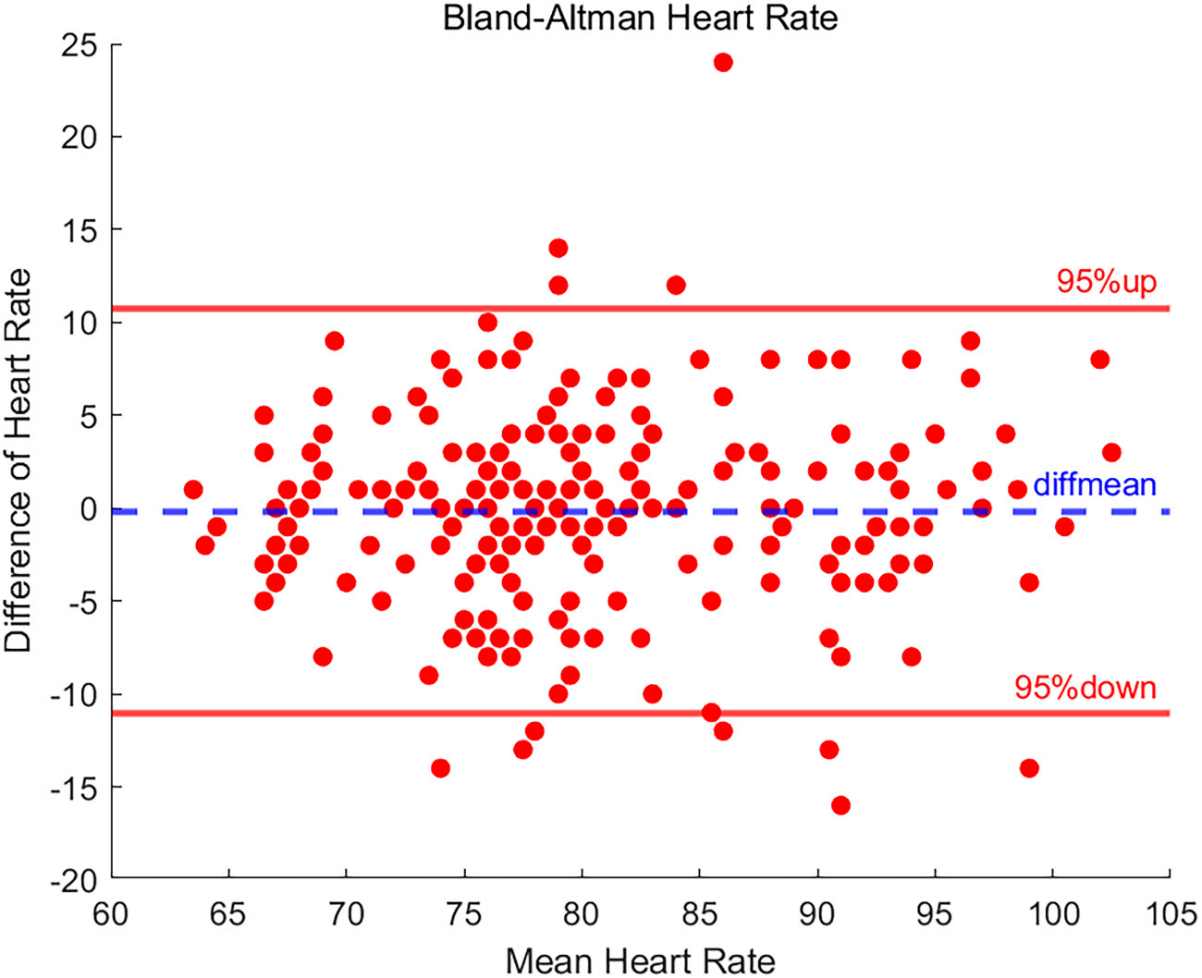
Bland-Altman plot of heart rate.

Analysis of the HR data using the Bland-Altman plot was shown in Figure 7. The calculation of the HR data showed an upper LOA interval of 10.736 and a lower LOA interval of −11.073.

The results indicated that 94.7% of the difference data fell within the 95% LOA, however, there were 11 sets of data where the differences exceeded the upper and lower limits of the consistency interval, which may indicate the presence of systematic error or external interference in the measurements.

The SD of the differences between the HR data was 5.563, with a mean difference of −0.168, which indicates a high degree of agreement between the HR data measured by the ES30 and the measurements of the BeneVision N15.94.7% of the data differences fell within the agreement interval indicating that these differences correspond to HR data measured by the EP30 device as compared to the BeneVision N15 with accuracy and consistency.

### Result of respiratory rate

**Figure 8.**
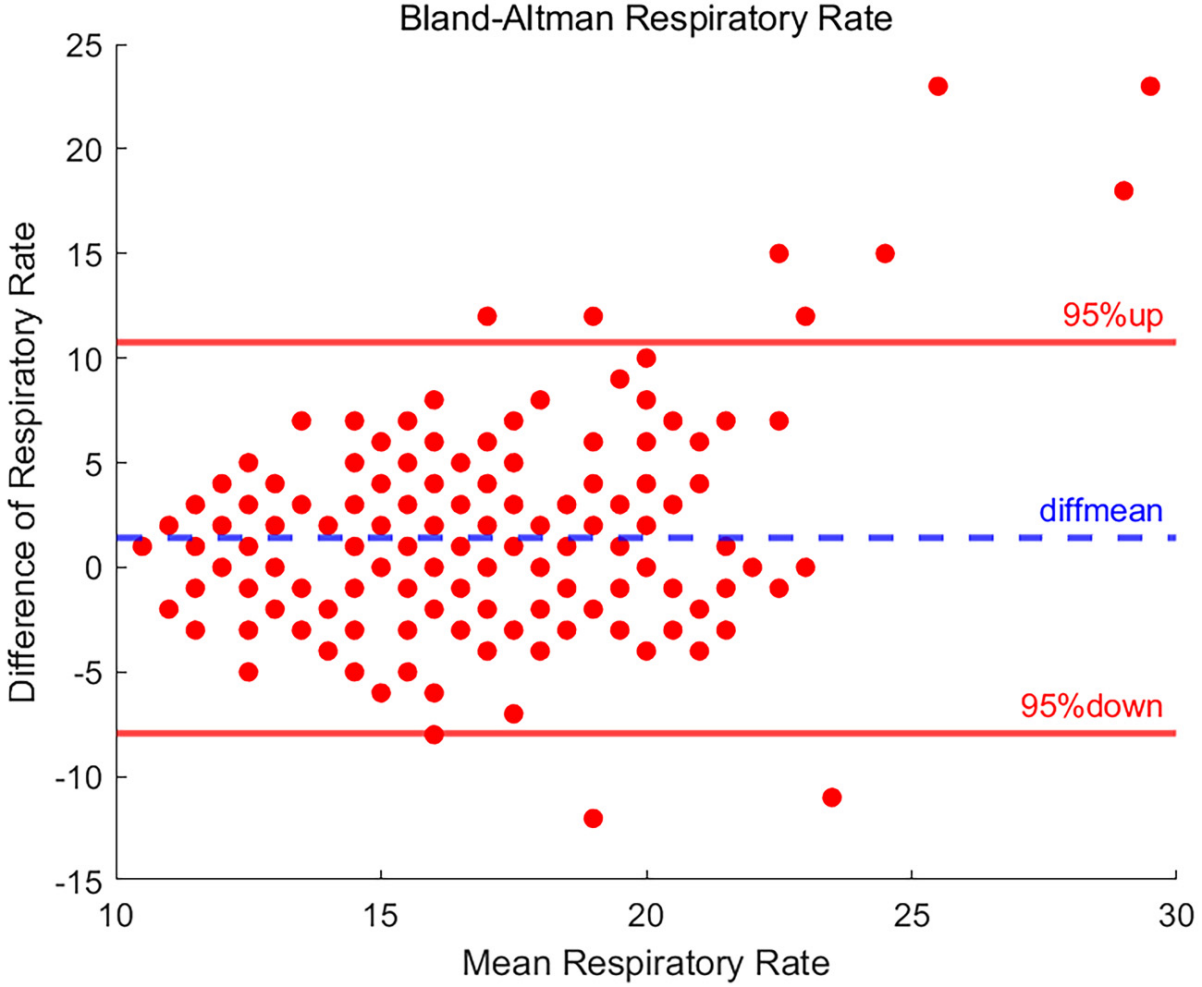
Bland-Altman plot of respiratory rate.

The results of the analysis of RRs were shown in Figure 8, with 94.7% of the data differences in RRs falling within the consistency interval.

However, there were 11 data points where the difference exceeded the upper and lower limits of the consistency interval, which may indicate that these data points may be outliers or due to some special circumstances (systematic error or external interference). The SD of the data for RR was 4.774 and the mean difference was 1.423, indicating that the ES30 measurements were slightly higher within the acceptable range compared to the BeneVision N15.

Bland-Altman analyses further revealed an upper LOA interval of 10.779 and a lower LOA interval of −7.933. These results indicate that the RR data measured by the ES30 device is in agreement with the reference value data provided by the BeneVision N15 device.

## Discussion and limitations

With ongoing advancements in medical technology, wearable vital monitoring devices are gaining increasing importance in clinical applications. In the future, their portability is likely to supersede traditional bedside vital monitoring systems. For some patients, this advantage enables real-time monitoring while they are on the move, a capability that bedside systems cannot provide.

For example, real-time monitoring of patients undergoing rehabilitation using a wearable device can significantly improve rehabilitation outcomes. The setting in which this device could provide benefit, is in community hospitals or at home. Monitoring of these patients with this device can be achieved from a distance, and potentially prevent patients being admitted for long-term stays in bigger general hospitals, away from their local area. This prevents unnecessary hospital admissions and the use of beds as fit enough patients can go home whilst being safely monitored.

This signals that healthcare services may rely more heavily on such devices in the future to provide more accurate and real-time health monitoring. This will help to improve the quality of life for patients, while also opening up new possibilities for healthcare services.

The study described in this article is to study the concurrent validity and comparability of wearable sensors in clinical environment. Therefore, we study both the simulator and real patients, and compare their performance with bedside monitors. This is a unique study undertaken to address concerns relating to the deployment of wearable devices in hospital. The BeneVision N15 device, which is widely used in clinical practice, was adopted for the purpose of this study as a reference device and was used to validate the comparability and agreement of the mWear device. This study employed the Bland-Altman analysis method, which is an important tool for assessing agreement between measurement techniques, to assess whether a wearable life monitoring device can be used as an alternative to bedside life monitoring devices.

In a pre-experiment using the ProSim 8 Simulator as a data source, it was indicated that the EP30 has similar stability and data accuracy as the BeneVision N15 when measuring the data provided by the simulator.

However, results indicated that the measurements taken by the various sub-devices of the mWear from the volunteer study showed slight deviations when compared to those of the more traditional bedside monitoring device, but generally indicated a high level of concurrent validity and consistency.

These results demonstrate the potential of the mWear device to replace traditional bedside vital monitoring devices in clinical settings, and could be used as a reference for clinical trials.

The results of the current study of the mWear device demonstrated its accuracy and concurrent validity, it has to be recognised that there are some limitations and shortcomings in the experiment. The source of data for this experiment was based on 16 healthy or sub-healthy volunteers, and although their data were authentic and reliable, their vital signs were not the same as those of various patients in the clinical setting, which may not fully represent the physiological diversity and conditions of clinical patients. Based on the experimental design, the volunteers were required to use two sets of devices simultaneously for data collection, and the BeneVision N15 device was not portable, and the volunteers were not able to perform other activities during the entire period of data collection, which resulted in the mWear device not reflecting its portability in the experiment.

Future research should focus on practical applications for clinical patients. The performance of wearable devices can be more fully evaluated by monitoring patients with different diseases, which can validate the adaptability of these devices to dynamic physiological conditions.

As the EP30 is like a watch, there may be reasons which limit their use due to the position. Firstly, if a patient has a cannula inserted at the wrist, it may cause discomfort for the patient. Secondly, the wristband of the EP30 is silicone, many patients may be allergic, causing pain and irritation. In addition, it may not be suitable for patients who have other skin conditions such as burns.

Ergonomics is another challenge wearables need to face. Some volunteers reported discomfort from the combined effect of the weight of the blood pressure cuff and the BP20 after 2 hours of data collection, which may be uncomfortable for patients when 24/7 data collection is required. At the same time, some female volunteers reported that the EP30's ring-type oximeter had a large lower limit of adjustment for diameter, which was still larger than the diameter of their finger when using the smallest diameter, which could lead to oximetry artefacts. This suggests the EP30 may have fitting limitations for individuals with slender fingers, such as some female or adolescent patients. Therefore, the design of wearable devices needs to focus not only on the functionality of the device, but also on the impact and effect of the device on the patient in practical use.

A limitation of this study is its focus on a single wearable system. While this allowed for an in-depth analysis, direct comparisons of the mWear device with other commercially available wearables were beyond the scope of this work and represent an important avenue for future research.

In addition to the above, the relatively small sample size (16 participants) limits the generalisability of our findings and may affect the statistical robustness of the results. The controlled experimental setting may also not fully reflect the complexity and variability of real-world clinical environments. Furthermore, the applicability of the mWear device to broader patient populations such as paediatric or elderly individuals remains to be explored. These aspects should be addressed in future studies with larger, more diverse populations and in more complex clinical scenarios.

## Conclusion

In conclusion, this work provides an insight into the agreement of wearable devices for clinical use and their substitutability for bedside vital monitoring devices, demonstrating the potential of the mWear device for clinical practice and the portability that allows patients to carry out freedom of movement. The Bland-Altman analytical methodology underpinned the present experiments, and taking the BeneVision N15 device's monitoring data as a reference, any systematic deviations or discrepancies between the data measured by the mWear device and the reference data could be detected. The results of this study emphasise the promising applications of the mWear device, but also the need for in-depth research into complex real-life use situations.

The results of this study showed a relative agreement of the mWear device's performance in clinical settings, with a high degree of consistency with traditional bedside monitoring systems. Its mobility and continuous monitoring ability give it an advantage in low-income countries or in healthcare organisations with limited resources. These results could also provide valuable insights and data to support the use of the mWear device in clinical settings.

While the experimental findings offered promise, additional validation is imperative, utilising monitoring data from actual patients with diverse health conditions, thereby addressing limitations observed during the experiment. Moreover, the ergonomic design process must prioritise patient-friendliness in real-life usage scenarios. Therefore, designers must have a profound understanding of patient needs to ensure that human factors, such as comfort, ease of use, and safety, are prioritised in future iterations. This patient-centric approach is essential for optimising the care and monitoring experience provided by the mWear device.
